# Mitochondrial ferritin, a new target for inhibiting neuronal tumor cell proliferation

**DOI:** 10.1007/s00018-014-1730-0

**Published:** 2014-09-12

**Authors:** Zhen-Hua Shi, Fang-Fang Shi, Yue-Qi Wang, Alex D. Sheftel, Guangjun Nie, Ya-Shuo Zhao, Lin-Hao You, Yu-Jing Gou, Xiang-Lin Duan, Bao-Lu Zhao, Hong-Meng Xu, Chun-Yan Li, Yan-Zhong Chang

**Affiliations:** 1 Key Laboratory of Animal Physiology, Biochemistry and Molecular Biology of Hebei Province, College of Life Science, Hebei Normal University, Shijiazhuang, 050024 Hebei China; 2 Key Laboratory of Molecular and Cellular Biology of Ministry of Education, College of Life Science, Hebei Normal University, Shijiazhuang, 050024 Hebei China; 3 Laboratory of Molecular Iron Metabolism, College of Life Science, Hebei Normal University, Shijiazhuang, 050024 Hebei China; 4 University of Ottawa Heart Institute, 40 Ruskin St., Ottawa, ON K1Y 4W7 Canada; 5 CAS Key Laboratory for Biomedical Effects of Nanomaterials and Nanosafety, National Center for Nanoscience and Technology of China, Beijing, 100190 China; 6 The Fourth Hospital of Hebei Medical University, Shijiazhuang, 050011 Hebei China; 7 The Second Hospital of Hebei Medical University, Shijiazhuang, 050000 Hebei China

**Keywords:** Neuroblastoma, Cyclin, Cell cycle, Cyclin-dependent protein kinase, Iron metabolism

## Abstract

Mitochondrial ferritin (FtMt) has a significant effect on the regulation of cytosolic and mitochondrial iron levels. However, because of the deficiency of iron regulatory elements (IRE) in FtMt’s gene sequence, the exact function of FtMt remains unclear. In the present study, we found that FtMt dramatically inhibited SH-SY5Y cell proliferation and tumor growth in nude mice. Interestingly, excess FtMt did not adversely affect the development of drosophila. Additionally, we found that the expression of FtMt in human normal brain tissue was significantly higher than that of neuroblastoma, but not higher than that of neurospongioma. However, the expression of transferrin receptor 1 is completely opposite. We therefore hypothesized that increased expression of FtMt may negatively affect the vitality of neuronal tumor cells. Therefore, we further investigated the underlying mechanisms of FtMt’s inhibitory effects on neuronal tumor cell proliferation. As expected, FtMt overexpression disturbed the iron homeostasis of tumor cells and significantly downregulated the expression of proliferating cell nuclear antigen. Moreover, FtMt affected cell cycle, causing G1/S arrest by modifying the expression of cyclinD1, cyclinE, Cdk2, Cdk4 and p21. Remarkably, FtMt strongly upregulated the expression of the tumor suppressors, p53 and N-myc downstream-regulated gene-1 (NDRG1), but dramatically decreased C-myc, N-myc and p-Rb levels. This study demonstrates for the first time a new role and mechanism for FtMt in the regulation of cell cycle. We thus propose FtMt as a new candidate target for inhibiting neuronal tumor cell proliferation. Appropriate regulation of FtMt expression may prevent tumor cell growth. Our study may provide a new strategy for neuronal cancer therapy.

**Electronic supplementary material:**

The online version of this article (doi:10.1007/s00018-014-1730-0) contains supplementary material, which is available to authorized users.

## Introduction

Neuroblastoma (NB) is one of the most severe pediatric cancers [1]. Although survival has been improved by recent therapies, NB is still one of the most difficult tumors to cure, with only 40 % long-term survival despite intensive multimodal therapy [2, 3]. While the past three decades have seen many advances, the elusive mechanisms of NB carcinogenesis make NB an enigmatic challenge to clinical and basic scientists. What is known about NB is that the amplification of the myc oncogene, a central player in many human cancers, dysregulates proliferation, apoptosis and differentiation, and is associated with poor prognosis [4, 5].

Much evidence has shown that iron (Fe) plays an important role in cell proliferation [6, 7]. In fact, tumor cells require more iron than normal cells to accommodate more rapid proliferation. Ribonucleotide reductase (RNR) is the rate-limiting enzyme involved in the conversion of ribonucleotides into deoxyribonucleotides (dNTPs) for DNA synthesis. The activity of RNR is dependent on Fe, since the enzyme complex’s R2 subunit contains a tyrosyl radical that requires Fe for stabilization. Congruent with this, it is well known that Fe depletion leads to G1/S arrest and apoptosis [8]. Additionally, iron chelation can cause hypophosphorylation of the retinoblastoma protein (pRb) by decreasing the expression of cyclins A, B and D, which are vital for cell cycle progression [9, 10]. Other regulatory molecules whose expression are affected by Fe depletion include p53, proliferating cell nuclear antigen (PCNA), Cdks, p21^CIP1/WAF1^ and hypoxia-inducible factor-1α (HIF-1α), which all take part in cell cycle regulation. While iron chelation can stimulate cell cycle arrest and apoptosis, on the other hand, iron excess can lead to an increased risk of developing cancer, presumably by the generation of reactive oxygen species [11]. In consideration of the above, iron may be considered a cofactor in tumor cell proliferation.

FtMt is an H-ferritin-like protein involved in modulating cellular iron metabolism [12–14]. Its physiological expression is restricted mainly to the testis, neuronal cells and islets of Langerhans [15, 16], while pathologically FtMt is highly expressed in ring sideroblasts [17]. Our previous studies and those of others have shown that FtMt is also involved in the regulation of oxidative stress [18, 19], but little is known about its exact function, especially in tumor tissue. Here, we show that the expression of FtMt is markedly decreased in nervous system tumoral tissue, including NB and neurospongioma (NS). Conversely, FtMt overexpression greatly suppresses SH-SY5Y neuroblastoma cells’ proliferation. We conclude that FtMt may be explored as a new target for inhibiting the proliferation of neuronal tumors.

## Materials and methods

### Methods

Dulbecco’s modified Eagle’s medium (DMEM), fetal calf serum and 4-(2-hydroxyethyl)-1-piperazineethanesulfonic acid (HEPES) were purchased from Gibco BRL (Grand Island, NY, USA). 3-(4,5-dimethylthiazol-2-yl)-2,5-diphenyl- tetrazolium-bromide (MTT), propidium iodide (PI), 5- or 6-(*N*-succinimidyloxycarbonyl)-3′,6′-O,O’-diacetylfluorescein (CFSE), penicillin and streptomycin were purchased from Sigma Chemical Co. (St. Louis, MO, USA). CellTrace™ CFSE Cell Proliferation Kit (C34554) was from Invitrogen (Shanghai, China). The plasmid of mouse mitochondrial ferritin and blank plasmid pcDNA3.1(−) were described previously [14]. The antibodies against HA (sc-805, 1:1,000 dilution), β-actin (sc-130656, 1:3,000 dilution), ferritin light chain (sc-390558, 1:1,000 dilution), pRb (sc-16670, 1:1,000), Rb (sc-50, 1:200 dilution), c-myc (sc-40, 1:500 dilution) and N-myc (bs-5980R, 1:200 dilution) were purchased from Santa Cruz Biotechnology (Santa Cruz, CA, USA). The antibodies against mitochondrial ferritin (ab124889, 1:1,000), ferritin heavy chain (H-ferritin) (ab65080, 1:5,000 dilution), p21 (ab109520, 1:1,000 dilution), cyclinD1 (ab101430, 1:1,000 dilution), lysine-specific demethylase 3A (JMJD1) (ab106456, 1:500 dilution), PCNA (ab140877, 1:1,000 dilution) and N-myc downstream-regulated gene-1 (NDRG1) (ab37897, 1:1,000 dilution) were from Abcam company (England). The antibodies against p53 (MS-105-P0, 1:1,000 dilution), Cdk2 (MS-617-P0, 1:500 dilution) and Cdk4 (MS-616-P0, 1:200 dilution) were from Thermo company (USA). The antibody against cyclinE (630701, 1:200 dilution) was from Biolegend (USA). The antibody against transferrin receptor 1 (TfR1) (1348053A, 1:2,000 dilution) was from Invitrogin (Shanghai, China). Human normal brain tissue (NBT), NB and NS tissue were from surgical operation patients. The patients involved gave their consent to this study, which had also been approved by the local ethics committee.

### Drosophila culture

Parents of four kinds of drosophilas (elav-Gal4, actin-Gal4, UAS-Fer3 HCH and wild-type W1118) were used to cross as shown in Table 1 [20, 21]. Each culture tube (diameter 2.5 cm, height 10 cm) had ten female and five male drosophilas (*n* = 8). When virgin drosophilas were selected, the older drosophilas of the culture tube were all removed and the newborn drosophilas within 10 h of hatching were collected. Then these new hatching drosophilas were subjected to carbon dioxide narcosis and female and male drosophilas were separated under stereology microscope. All drosophilas were cultured at a humidity of 60 % at 25 °C for 12 h under light or dark condition, respectively. After culturing for 3 days, the parent drosophila were removed. The number of F_1_ generation cultured 7–10 days was analyzed.

### Cell culture

SH-SY5Y, FtMt-transfected cells (FtMt-SY5Y) and vector pcDNA3.1-transfected cells (pcDNA3.1-SY5Y) [18] were maintained in DMEM supplemented with heat-inactivated fetal calf serum (10 %, vol/vol), glucose (4.5 mg/ml), penicillin (100 U/ml) and streptomycin (100 mg/ml) in humidified 5 % CO_2_ and 95 % air at 37 °C. pcDNA3.1-SY5Y and FtMt-SY5Y cells were maintained in G418 800 µg/ml to select stable FtMt-transformed SH-SY5Y cells. Where indicated, cells were cultured in the presence of ferric ammonium citrate (FAC).

### Xenograft tumor growth in nude mouse

Male athymic Balb/c nu/nu mice, 4 weeks of age and specific pathogen free were obtained from Vital River Laboratories (Beijing, China). Mice were housed in microisolator cages with autoclaved bedding in a specific pathogen-free facility with 12-h light–dark cycles. Animals received pathogen-free water and food ad libitum. Mice were inoculated with 1.5 × 10^7^ cells/ml in 0.2 ml phosphate-buffered saline (PBS) subcutaneously. Tumor growth state was observed weekly after the tumors become visible. 8 weeks after injection, mice were humanely killed and the primary tumor volumes and weights were measured, respectively.

### Quantitative real-time PCR (qRT-PCR)

The quantity of p21 gene mRNA expression was detected by qRT-PCR (using SYBR Green) with an Applied Biosystem 7500 Fast Real-time PCR System. The primer sequences used for the qRT-PCR reaction were as follows: p21 (178 bp) sense p21: TGGACCTGTCACTGTCTTGT and antisense p21: TCCTGTGGGCGGATTAG. All PCRs were performed in triplicate.

### Assessment of cell proliferation

Cell proliferation was measured by MTT assay according to the literature [22] and 5- or 6-(*N*-succinimidyloxycarbonyl)-3′,6′-*O*,*O*’-diacetylfluorescein (CFSE) labeling [23]. In brief, exponentially growing SH-SY5Y cells, FtMt-SY5Y cells, or pcDNA3.1-SY5Y cells were harvested with 0.25 % trypsin–0.02 % EDTA and then plated at a density of 1 × 10^4^/well in 96-well plates. After incubation for the indicated times, cell viability was determined by adding MTT (500 mg/ml) to each well, and the mixture was incubated for another 4 h at 37 °C. After the medium was removed, cells were lysed with DMSO. The absorbance at 595 nm was measured with a Bio-Rad model 3550 microplate reader (Richmond, CA, USA). The samples were measured in eight replicates, and each experiment was repeated three times.

CFSE is widely used for cell proliferation assays [24]. The CFSE labeling assay was performed using the CellTrace™ CFSE Cell Proliferation Kit (C34554) according to the manufacturer’s instructions. Briefly, cells were suspended in PBS at a final concentration of 1 × 10^6^/ml, to which 5 mM of stock CFSE solution was added to achieve a final working concentration of 10 µM and incubated at 37 °C for 10 min. The staining was halted by the addition of 5 volumes of ice-cold culture media to the cells and incubation for 5 min on ice. Cells were pelleted by centrifugation and washed with fresh media three times. Cells were then cultured for 24 h and finally harvested and analyzed by flow cytometry.

### Detection of apoptosis by propidium iodide staining

The influence of FtMt on apoptosis in SH-SY5Y cells was measured using a propidium iodide (PI) kit according to the manufacturer’s instruction. Briefly, the cells were plated at a density of 5 × 10^5^ in 60-mm cell culture dishes and incubated overnight for cell attachment. The exponentially growing cells were incubated for 24 h, at which time they were harvested and washed three times with cold PBS and resuspended in 500 µl of binding buffer. The cell suspension was incubated in the dark for 15 min with 5 µl of PI staining solution. Quantification of labeling was determined by flow cytometry using a BD FACSCalibur flow cytometer (BD Biosciences, Bedford, MA, USA).

### Cell cycle analysis

Cell cycle analysis was performed using GenScript’s Cell Cycle Analysis Kit according to the manufacturer’s instructions. Briefly, the cells were cultured for 24 h and then harvested, washing cells once in an excess of PBS. Cells were then centrifuged at 0.3×*g* for 5 min and the supernatant discarded. The cells were resuspended in PBS to a titer of 1 × 10^6^/ml. One million cells from this suspension were then pelleted and resuspended in 500 μl of 70 % (v/v) ice-cold ethanol for 2 h. After washing the cells in PBS, cells were resuspended in 100 μl of PBS and incubated at 37 °C for 30 min. PI solution (400 μl) was then added, followed by a 30-min incubation at 4 °C, in the dark. PI staining was determined by flow cytometry on a FACSCalibur flow cytometer, and data were analyzed by Cell Quest^®^ software.

### Estimation of the intracellular labile iron pool

The intracellular labile iron pool (LIP) was assayed as previously described [18], with some modification. Briefly, cells in exponential growth were harvested, washed three times with PBS and resuspended in buffer CA (140 mM NaCl, 5 mM KCl, 1 mM MgCl_2_, 5.6 mM glucose, 1.5 mM CaCl_2_, 20 mM HEPES, pH 7.4). Calcein AM (final concentration 0.25 µM) was then added and the reaction mixture incubated for 30 min at 37 °C. After washing three times, the cells were resuspended in buffer CA and transferred to a fluorometer cuvette. The fluorescence intensity of calcein AM was followed by continuous acquisition in a fluorescence spectrophotometer (Hitachi F-4500), at an excitation wavelength of 485 nm and an emission wavelength of 520 nm. Once a stable baseline was achieved, salicylaldehyde isonicotinoyl hydrazone (SIH) (final concentration 100 µM) was added and the increase in fluorescence was used to estimate the levels of calcein-bound iron.

### Immunofluorescence microscopy

Immunocytochemical studies were performed as described previously [25]. Cells were washed with PBS and fixed in 3.7 % formaldehyde (in PBS) for 20 min at 4 °C. Cells were permeabilized with PBS containing 0.2 % Triton X-100 for 5 min, blocked with 5 % BSA for 1 h and then washed three times with PBS. Incubation with primary antibody was carried out for 1 h at room temperature. Excess antibody was removed by washing three times with PBS. This was followed by incubation with an appropriate fluorophore-labeled secondary antibody for 1 h at room temperature in an area protected from light. After removing excess antibody by washing three times with PBS, mounting was performed using a ProLong Antifade Kit (Invitrogen). Images were obtained by fluorescence microscopy (Axio Imager M1; Zeiss, Oberkochen, Germany).

### Western blotting

The method of Western blotting has been described previously by Shi et al. [18]. Proteins were extracted from fresh-frozen tissues and cultured cells. Briefly, the tissues were homogenized and lysed with RIPA buffer containing 100 µg/ml PMSF and 1 µg/ml aprotinin. The lysate was collected, kept on ice for 15 min and centrifuged at 12,000×*g* at 4 °C for 10 min. Equal amounts of protein (30–50 μg) were loaded and separated by SDS-PAGE. For cultured cells, the samples were washed twice with cold PBS, lysed in lysis buffer (50 mM Tris–Cl, 150 mM NaCl 0.02 % NaN_3_, 100 μg/ml PMSF, 1 μg/ml aprotinin, 1 μg/ml pepstatin A, 2 μg/ml leupeptin, 1 % Triton X-100) on ice for 30 min and then sonicated for 3 × 10 s. After centrifugation at 12,000*g* for 30 min at 4 °C, the supernatant was collected. Protein content was estimated by a BCA assay kit (Pierce Biotechnology). Forty micrograms of protein from each sample was resolved by SDS-PAGE and then transferred to nitrocellulose membranes. Blots were blocked in blocking buffer containing 5 % fat-free milk and 0.1 % Tween 20 in 0.1 M tris buffered saline (TBS) and incubated with a primary antibody overnight with constant agitation at 4 °C. After washing four times, the membranes were incubated with a secondary antibody for 1 h at room temperature with constant agitation, then washed, treated with a chemiluminescence substrate (Pierce Biotechnology, IL, USA) and exposed to Kodak-XAR film. The developed film was digitized and analyzed by ImageJ (NIH, Bethesda, USA).

### Statistical analysis

All experiments were performed at least three times. One-way ANOVA was used to estimate the overall significance determined by Tukey’s tests corrected for multiple comparisons. Data are presented as mean ± SD. A probability level of 5 % (*p* < 0.05) was considered to be significant.

## Results

### Expression of FtMt and TfR1 in nervous tissue tumor

**Fig. 1:**
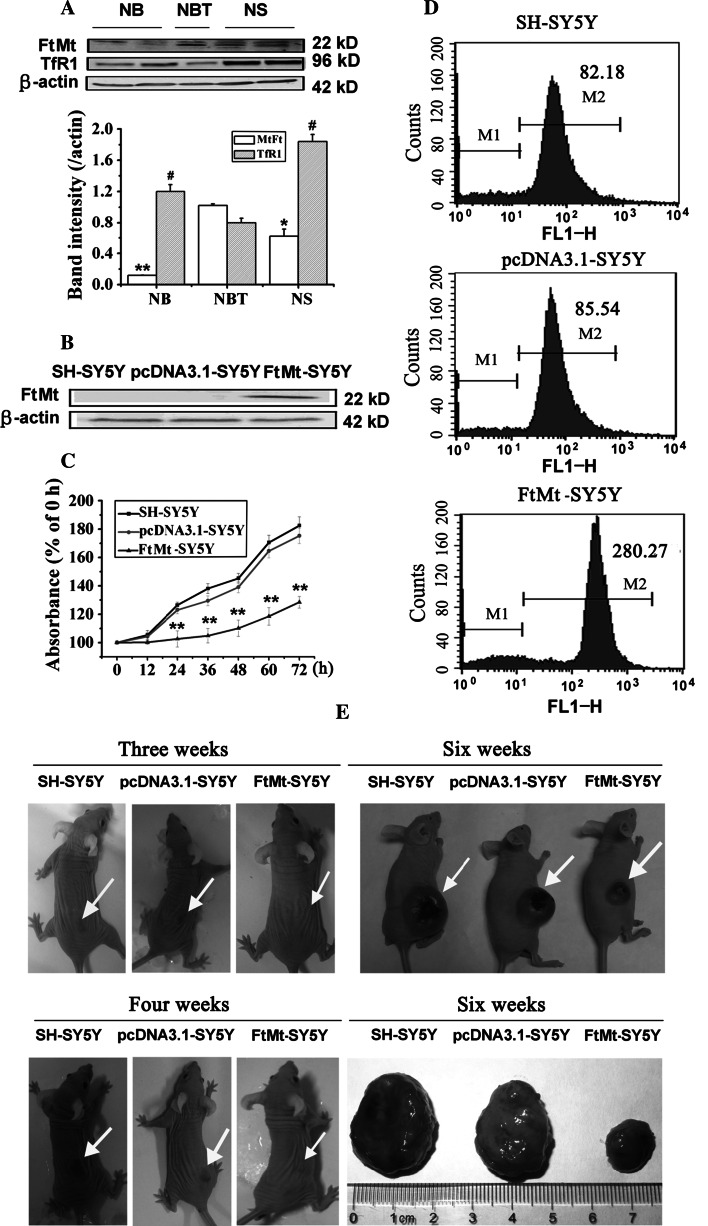
The expression of FtMt and TfR1 in tumor tissues and effect of FtMt on cell proliferation in SH-SY5Y cells. **a** The expression of FtMt and TfR1 in NB and NS. Tumor tissues were extracted and subjected to Western blot analysis (**a**, *upper panel*) and quantification thereof (**a**, *lower panel*) as described in “Methods”; data are shown as mean ± SD, *n* = 3, **p < 0.01 vs. NBT group, *p < 0.01 and ^#^ *p* < 0.05 vs. NBT group. **b** Expression of FtMt in stable SH-SY5Y cell lines as determined by Western blot. **c**, **d** Cell growth was determined by MTT and flow cytometry labeled with CFSE. Cells were cultured for 24 h, then harvested and processed; data were the average of three experiments. Data were expressed as percentage of cell growth compared with control cells ± SD, *n* = 3, **p < 0.01 vs. control group. **e** Photo of visible tumors (*arrow*) at 3, 4 and 6 weeks post-cell implantation in nude mice. The *lower right panel* exhibits the volume and weight of the tumors excised at 6 weeks

To identify the role of FtMt in nervous tissue tumors, the expression of FtMt and TfR1 in NB and NS was estimated. Interestingly, FtMt protein levels were significantly lower, while those of TfR1 were elevated in NB and NS tissues, when compared with NBT (Fig. 1a). The negative correlation between TfR1 and FtMt may suggest that FtMt plays an important role in iron metabolism and cancer cell proliferation.

### Mitochondrial ferritin overexpression inhibits neuronal tumor cell proliferation without initiating apoptosis

Based on the above results, we hypothesized that FtMt upregulation could inhibit the proliferation of neuroblastoma cells. To test this, we manipulated the levels of FtMt in the NB cell line, SH-SY5Y [18] (Fig. 1b), and then estimated the growth of the cells by MTT assay or CFSE labeling. The proliferation rate of FtMt-overexpressing SH-SY5Y (FtMt-SY5Y) cells from 24 to 72 h was much slower than that of SH-SY5Y and vector only (pcDNA3.1-SY5Y) cells, when the cells were cultured consecutively for the indicated times (Fig. 1c). To further examine the effects of FtMt on cell proliferation, we used CFSE labeling, followed by flow cytometry. As shown in Fig. 1d, when the cells were cultured for 24 h prior to labeling, the fluorescence intensities (in arbitrary units) of FtMt-SY5Y, pcDNA3.1-SY5Y and SH-SY5Y cells were 280.27, 85.54 and 82.18, respectively. This fourfold increase in signal from FtMt-overexpressing cells indicates that FtMt overexpression strongly inhibits tumor cell division.

**Fig. 2:**
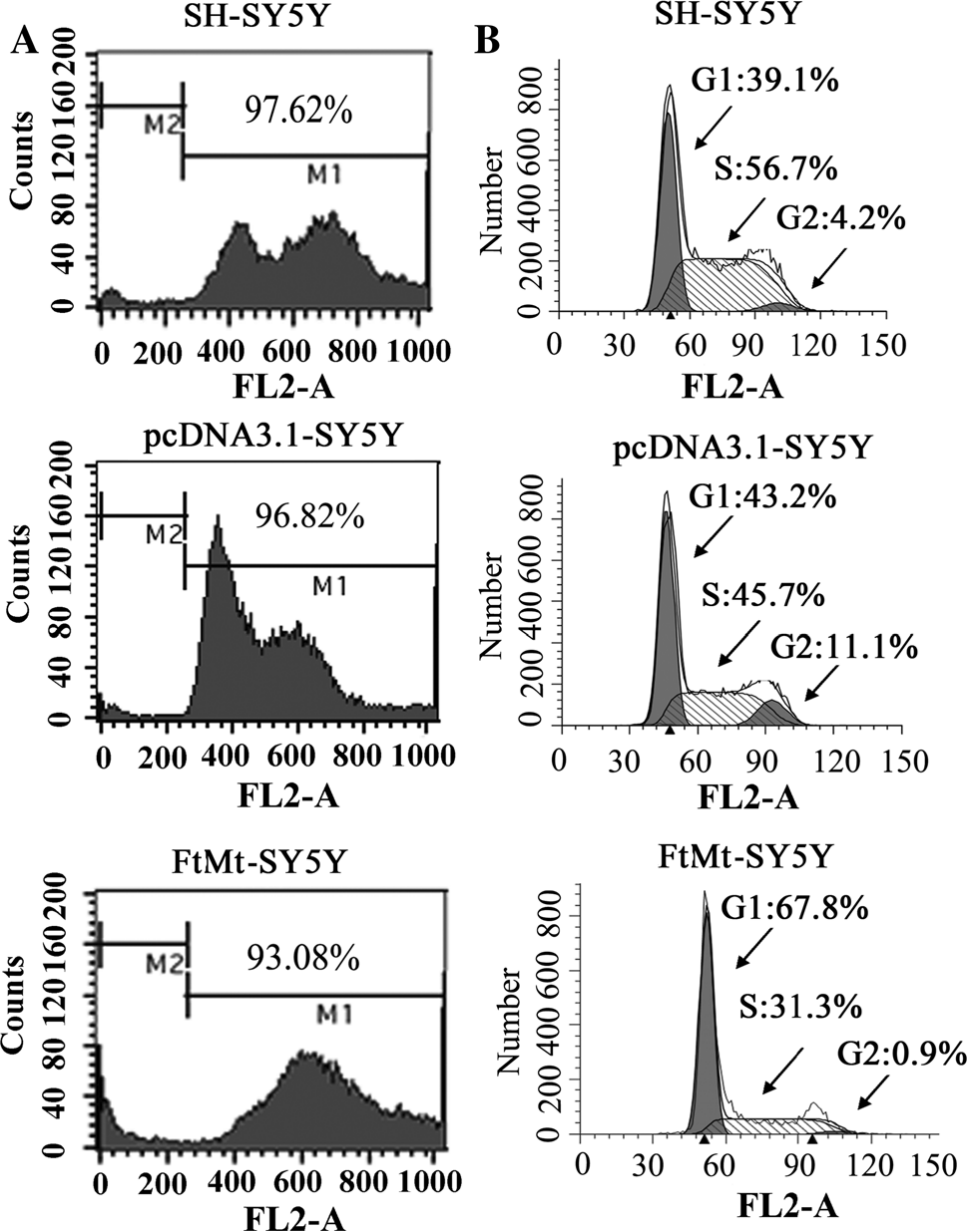
The effect of FtMt overexpression on apoptosis and cell cycle. **a**, **b** Apoptosis and **c** cell cycle were examined by flow cytometry of cells labeled with PI. For apoptosis and cell cycle determination, cells were cultured for 24 h, then harvested and processed. Data shown are representative of three experiments

To further observe the tumor cells; growth state, we subcutaneously implanted cells into nude mice. As shown in Fig. 1e, when the cells were transplanted for 3, 4 and 6 (Fig. 1e) weeks, the tumors from SH-SY5Y and pcDNA3.1-SY5Y were visible and became large with time, while tumors derived from FtMt-SY5Y cells were considerably smaller than those of control groups. The average weight of tumors from SH-SY5Y and pcDNA3.1-SY5Y cells were 2.52 ± 0.2 g and 2.13 ± 0.19 g, respectively, while, the average weight of tumors from FtMt-SY5Y was only 0.45 ± 0.031 g. To verify that the FtMt indeed caused apoptosis in the SH-SY5Y cells, resulting in decreased MTT signal, the effect of FtMt overexpression on apoptosis was measured by flow cytometry. Surprisingly, as shown in Fig. 2a, the apoptosis rates of SH-SY5Y, pcDNA3.1-SY5Y and FtMt-SY5Y cells were (2.38 ± 0.213) %, (3.18 ± 0.271) % and (6.92 ± 0.429) %, respectively, when the cells were cultured for 24 h, indicating that FtMt expression has little influence on apoptosis in SH-SY5Y cells.

To investigate the effects of FtMt on normal cell growth, we examined the development of drosophila overexpressing FtMt in nervous tissue alone or in all tissues. As shown in Table 1, whether in the nervous system alone or ubiquitously, overexpression of FtMt had no effect on F_1_ generation development. Thus, overexpression of FtMt did not affect normal tissue growth and development.

**Table 1:** The effect of FtMt on the development of drosophila F_1_ generation

Group (*n* = 8)	W1118/W1118 (number of F_1_ generations flies)	Actin-Gal4/UAS-Fer3 HCH (number of F_1_ generations flies)	Elav-Gal4/UAS-Fer3 HCH (number of F_1_ generations flies)
1	121	123	108
2	89	48	105
3	70	76	101
4	92	46	104
5	96	38	115
6	98	152	127
7	99	105	97
8	131	121	133

W1118/W1118: F_1_ generations are all wild type; actin-Gal4/UAS-Fer3 HCH: half of F_1_ generations are overexpression of FtMt in all tissues of drosophila, another half F_1_ generations are wild type in all tissues of drosophila; elav-Gal4/UAS-Fer3 HCH: all of F1 generations are overexpression of FtMt only in nerve tissue. At the 0.01 level, the population means are not significantly different among the three groups

### Excess mitochondrial ferritin arrests the cell cycle at the G1/S transition

Cell proliferation depends on a continuous cell cycle. To investigate the effects of FtMt on SH-SY5Y cell growth, flow cytometry of PI-labeled cells was performed [26]. The numbers of cells in G1 and S phases in SH-SY5Y cells and pcDNA3.1 cells were 39.1 and 56.7 % and 43.2 and 45.7 %, respectively, while those of FtMt-SY5Y cells were 67.8 and 31.3 % (Fig. 2b). These results show that elevated FtMt inhibits the growth of SH-SY5Y neuroblastoma cells by arresting the cell cycle.

### The effects of elevated FtMt on the expression of cyclins and cyclin-dependent protein kinases

**Fig. 3:**
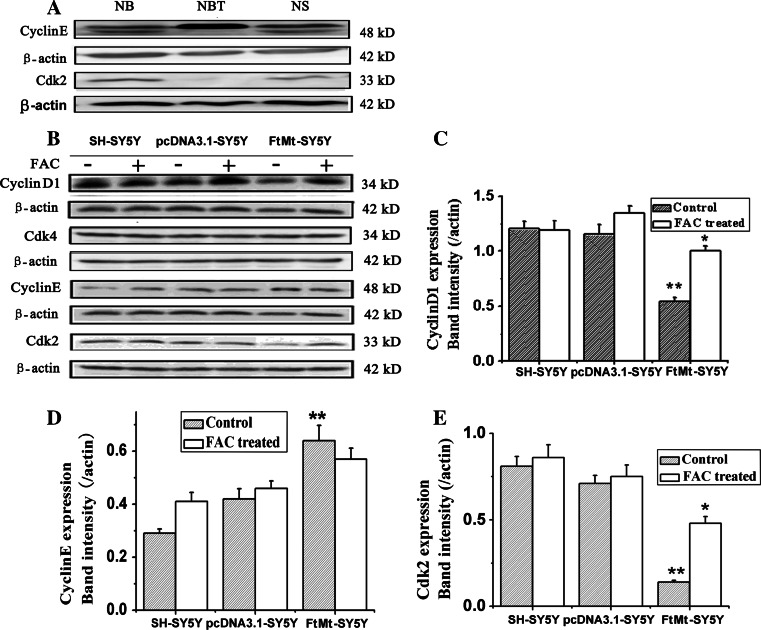
The expression of related cyclins and Cdks in NB and NS and the effect of FtMt on these proteins in cultured cells. **a** The relative expression of cyclinE and Cdk2 in NB and NS was estimated by Western blot analysis. **b** The expression of cyclinD1, Cdk4, Cdk2 and cyclinE in SH-SY5Y, pcDNA3.1-SY5Y and FtMt-SY5Y cells. Where indicated, samples were treated with (+) or without (−) 100 μM FAC for 24 h. Data are shown as mean ± SD, *n* = 3, **p < 0.01 vs. control group, *p < 0.01 vs. FAC-treated FtMt cells

The cyclins are a family of proteins that control the progression of cells through the cell cycle by activating cyclin-dependent kinases (Cdks) [27]. Cyclins themselves have no enzymatic activity, but have binding sites for specific substrates and thus recruit Cdks to specific subcellular locations. Cyclins can be divided into four classes based on their behavior in the cell cycle, with different cyclin classes having roles in specific segments of the cell cycle. In general, without a corresponding cyclin, a Cdk has little kinase activity: only the cyclin–Cdk (such as cyclinE–Cdk2, cyclin D1–Cdk4) complex is an active kinase. Since elevated FtMt resulted in cell cycle arrest at the G1/S phase, we tested the relative expression levels of G1/S arrest-related cyclins and Cdks in tumor tissues and cultured cells. As shown in Fig. 3a, the expression of cyclinE was upregulated and Cdk2 was downregulated significantly in NBT compared with tumor tissues. Meanwhile, overexpression of FtMt caused the expression of both cyclinD1 and Cdk2 to decrease significantly, while the expression of Cdk4 exhibited little change and CyclinE increased slightly (Fig. 3b). Although the upregulation of cyclinE may be expected to permit cell cycle progression, the strong decreases in both cyclinD1 and Cdk2, following FtMt overexpression were likely the cause of G1/S arrest.

### FtMt inhibits cell growth through the alteration of cellular iron distribution

Following its overexpression, FtMt has been shown to result in cytoplasmic iron deficiency [18, 28]. To test whether the inhibition of cell cycle observed in cells with elevated FtMt is related to iron, we attempted to rescue the cell cycle arrest by adding exogenous iron salts. The three SH-SY5Y stable cell lines were incubated with 100 µM ammonium ferric citrate (FAC) for 24 h and then examined for changes in cyclin/Cdk levels. Adding iron upregulated the expression of Cdk2 and cyclinD1, but had little influence on the expression of cyclinE and Cdk4, as shown in Fig. 3b. These results suggest that FtMt can arrest the cell cycle by its mitochondrial iron sequestration activity.

**Fig. 4:**
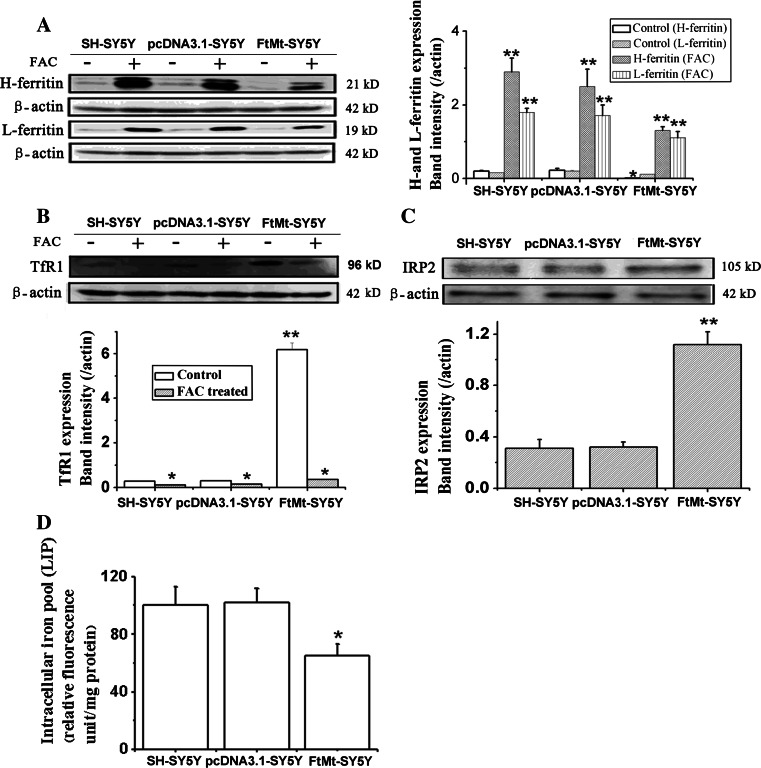
The effect of FtMt overexpression on proteins of cellular iron metabolism and the LIP. **a** The expression of H-ferritin and L-ferritin in SH-SY5Y, pcDNA3.1-SY5Y and FtMt-SY5Y cells with (+) or without (−) FAC. Data are shown as mean ± SD, *n* = 3, **p < 0.01 vs. control group, *p < 0.05 vs. control group. **b** The expression of TfR1, **c** the expression of IRP2 in SH-SY5Y, pcDNA3.1-SY5Y and FtMt-SY5Y cells with (+) or without (−) FAC. **d** LIP was detected using calcein AM as described in “Methods”. Data are shown as mean ± SD, *n* = 3, **p < 0.01 vs. control group, *p < 0.05 vs. control group and *p < 0.01 vs. FAC-treated FtMt cells

Iron is an essential component of many proteins and enzymes that are involved in cell growth and replication by regulation of the cell cycle pathway [9]. Ferritin, transferrin receptor 1 (TfR1), IRP2 and the labile iron loop (LIP) are four factors that can directly reflect intracellular iron levels. Our previous study shows that FtMt can sequester intracellular iron and accordingly protect cells from apoptosis by iron-induced oxidative stress [18]. Here, we investigated whether FtMt inhibited cell growth and regulated cell cycle by limiting the amount of iron available for normal cell function. The expression of H-ferritin and L-ferritin in control or FtMt-overexpressing cells, with or without the addition of FAC, was determined by Western blot analysis. As shown in Fig. 4a–c, elevated FtMt caused a decrease in the expression of H-ferritin and L-ferritin, while increasing TfR1 and IRP2, compared with control samples. These data indicate that FtMt can induce a cellular iron deficiency response. We were able to rescue the observed iron deficiency phenotype by supplying exogenous iron: when the cells were treated with FAC, the expression of H-ferritin and L-ferritin increased significantly, as TfR1 markedly decreased. Further, as shown in Fig. 4d, the level of iron in the LIP in FtMt-SY5Y cells was significantly lower than that of the control group. Taken together, our results show that cytosolic iron deficiency induced by FtMt overexpression could elicit the observed anti-proliferation phenotype.

### FtMt overexpression affects the expression of cell proliferation regulators (PCNA, p53, Rb and p21)

**Fig. 5:**
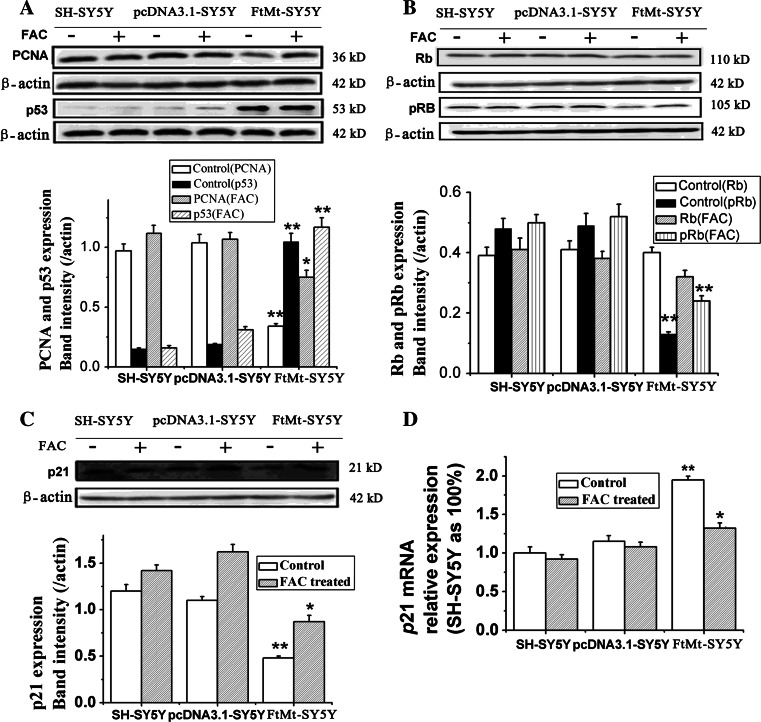
The effects of FtMt overexpression on PCNA, P53, pRb/Rb and p21. **a** The protein levels of PCNA and p53, **b** pRb/Rb and **c** p21 in SH-SY5Y, pcDNA3.1-SY5Y and FtMt-SY5Y cells treated with (+) or without (−) FAC were determined by Western blot analysis. **d** p21 mRNA levels were also determined by qRT-PCR. Data are shown as mean ± SD, *n* = 3, **p < 0.01 vs. control group, *p < 0.05 vs. control group

Unchecked cancer cell proliferation arises as a result of multiple factors. In addition to the cyclins and Cdks, other proteins including Rb, p53, p21 and PCNA play important roles in cell proliferation. PCNA is an auxiliary protein necessary for DNA synthesis [29] and is used as a marker of cell proliferation in tissues to assess the efficiency of chemotherapeutic drugs [30, 31]. p53 is crucial in regulating the cell cycle and functions as a tumor suppressor that is involved in preventing oncogenesis [32, 33]. Rb is another tumor suppressor protein that is dysfunctional in several cancers [34]. In the hypophosphorylated state (p-Rb), Rb is active and carries out its role as a tumor suppressor by inhibiting cell cycle progression. p21 is a potent cyclin-dependent kinase inhibitor which binds to and inhibits the activity of cyclin–Cdk2 or –Cdk1 complexes, thus functioning as a regulator of cell cycle progression at G1/S [35]. To evaluate if the growth-inhibitory effects of elevated FtMt on tumor may be through these regulator proteins, we tested the expression of these proteins or genes by Western blot analysis and qRT-PCR, respectively. As shown in Fig. 5a, overexpression of FtMt markedly downregulated PCNA, while increasing p53 protein levels. In fact, the level of p53 was sevenfold higher in FtMt-overexpressing cells compared to controls. In addition, the level of phosphorylated Rb (p-Rb) significantly decreased upon increased FtMt expression. Interestingly, the expression changes of these proteins were partially reversed by treatment with FAC (Fig. 5a, b). These results indicate that FtMt overexpression inhibited cell growth through the PCNA, p53 and Rb pathways through a mechanism in which iron plays some role.

In addition to cyclins and Cdks, cyclin-dependent kinase inhibitors also play important roles in cell cycle control. p21 is a cyclin-dependent kinase inhibitor that binds to and abrogates the activity of cyclin–Cdk2 complexes, thus functioning as a regulator of cell cycle progression at G1. The expression of this gene is tightly controlled by the tumor suppressor protein p53, through which p21 mediates p53-dependent, cell cycle G1 phase arrest in response to a variety of stress stimuli. To test p21′s role in the cell cycle under the overexpression of FtMt, p21 protein and mRNA levels were assayed by Western blot analysis and qRT-PCR, respectively. The expression of p21 protein markedly decreased compared to control (Fig. 5c; *p* < 0.05), even though the mRNA level increased significantly (Fig. 5d; *p* < 0.01). Again, supplemental iron could partially recover these effects of FtMt overexpression (Fig. 5c). This observation is consistent with a previous study in which iron chelation upregulated p21^CIP1/WAF1^ mRNA, but paradoxically inhibited its translation [22, 36]. In consideration of this, together with our results, it seems likely that the iron-sequestering property of FtMt mediates its effects on the cell cycle.

### The effect of FtMt overexpression on the levels of c-myc, N-myc, NDRG1 and JMJD1

**Fig. 6:**
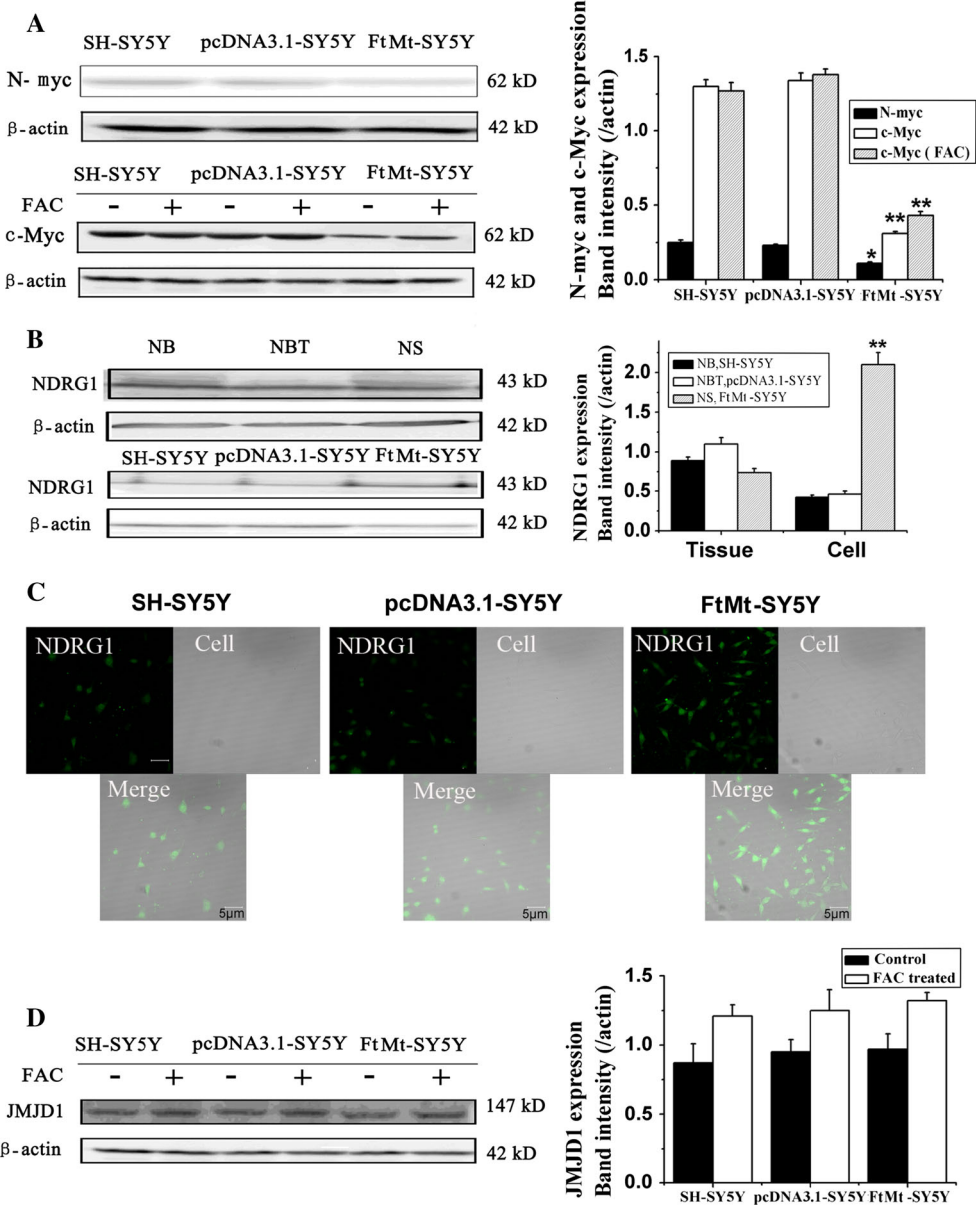
The effects of elevated FtMt on N-myc, c-myc, JMJD1 and NDRG1 expression in tumor tissue and cells. **a** The expression of N-myc and c-myc in SH-SY5Y, pcDNA3.1-SY5Y and FtMt-SY5Y cells by Western blot analysis. **b** The expression of NDRG1 in NB, NS and culture cells was determined by Western blot analysis. **c** Assay of NDRG1 in SH-SY5Y, pcDNA3.1-SY5Y and FtMt-SY5Y cells by immunohistochemistry. **d** The expression of JMJD1 in SH-SY5Y, pcDNA3.1-SY5Y and FtMt-SY5Y cells. Data are shown as mean ± SD, *n* = 3, **p < 0.01 vs. control group, *p < 0.05 vs. control group and *p < 0.01 vs. FAC-treated FtMt cells

c-myc is one of the myc family of transcription factors which activates the expression of a myriad of genes by binding to consensus sequences and recruiting histone acetyltransferases. c-myc can also act as a transcriptional repressor and has a direct role in the control of DNA replication [37]. A potent proto-oncogene, c-myc, is often found to be upregulated in many types of cancers. c-myc overexpression stimulates gene amplification [38], presumably through DNA over-replication, which can have a profound effect on the control of cell growth. Interestingly, it has already been demonstrated that c-myc overexpression predisposes cells to iron homeostasis disruption [39]. To elucidate whether the effects of elevated FtMt on cell growth inhibition may operate through the Myc pathway, we measured the expression of c-myc and N-myc by Western blot analysis. As shown in Fig. 6a, overexpression of FtMt significantly downregulated the expression of c-myc and N-myc when compared with controls (*p* < 0.01). When the cells were treated with FAC for 24 h, the expression of c-myc in FtMt-SH-SY5Y cells slightly recovered, while FAC treatment had no effect on c-myc levels in SH-SY5Y and pcDNA3.1-SY5Y cells. Our data suggest that increased FtMt halts cell proliferation through the c-myc and N-myc pathway in an iron-dependent manner.

It has been reported that NDRG1 is involved in tumor metastasis and, hence, negatively correlates with tumor progression in multiple neoplasms. Our result show that NDRG1 is upregulated slightly in NBT compared with NB and NS, but NDRG1 levels increase significantly in FtMt-SY5Y cells compared with controls whether by Western blot or immunohistochemistry assay (Fig. 6b, c), indicating that elevated FtMt may inhibit tumor cell metastasis via NDRG1.

A number of histone demethylases, whose biological functions remain largely uncharacterized, have been identified. These enzymes are often found to be associated with malignancy. JMJD1 belongs to a family of histone demethylases, which are iron- and 2-oxoglutarate dependent [40]. JMJD1 is a positive regulator of the G1/S transition in cancer cells [41]. The contribution of JMJD1 deregulation to oncogenesis has been hinted at in a couple of previous reports that correlated JMJD1 abnormalities with tumorigenesis [42, 43]. Since JMJD1 is iron dependent and FtMt overexpression decreases LIP levels (see above), we tested whether FtMt had an effect on the levels of JMJD1. As shown in Fig. 6d, there is no difference in the expression of JMJD1 among the three cell lines and iron has no effect on JMJD1 levels, suggesting that JMJD1 does not play a role in neuroblastoma oncogenesis.

## Discussion

Iron (Fe) is an absolute requirement for life. For instance, Fe plays a crucial role in the conversion of ribonucleotides into deoxyribonucleotides as an obligate cofactor in the rate-limiting step of DNA synthesis catalyzed by the chemotherapeutic target, RNR. It is therefore not surprising that, without Fe, cells are unable to proceed from the G1 to the S phase of the cell cycle [44]. On the other hand, PCNA, a marker of S phase and an auxiliary protein of DNA polymerase δ [45, 46], was significantly increased in iron-loaded livers, suggesting enhanced proliferation. Several studies have shown that iron levels are connected to the carcinogenic process [47–49]. Furthermore, it is well known that Fe depletion leads to G1/S arrest and apoptosis and Fe chelators can inhibit the growth of aggressive tumors [50, 51]. In fact, the clinically used chelator, desferrioxamine (DFO), is capable of potent cytotoxic effects on neuroblastoma cells, not only in vitro but also in clinical trials, underscoring the strategy of Fe deprivation as a viable therapeutic approach [52, 53]. Despite the well-known connection of Fe to cell proliferation and DNA synthesis [54–57], surprisingly little is known about the mechanism of iron’s involvement in these processes, especially in the molecular control of cell cycle progression in tumor tissues. In our study, we have forced the sequestration of iron in mitochondrial ferritin to evaluate the molecular consequences of cytosolic and nuclear iron starvation in neoplastic cells.

FtMt is an H-ferritin-like protein that can affect cellular iron metabolism [12–14]. This protein’s physiological expression is restricted mainly to the testis, but is also observed in neuronal cells. Unlike cytosolic ferritin, whose mRNA harbors an iron response element, FtMt is not subject to the classical, post-transcriptional regulatory system known as the IRE–IRP pathway (for a review see [58]). So far, the exact function of FtMt is not clear, in normal or tumor cells.

Our study is the first to evaluate the expression of FtMt in NB and NS tissues. We found that FtMt was significantly downregulated in NB and NS tissues compared with normal neuronal tissue. At the same time, TfR1 was markedly elevated in tumors (Fig. 1a), which, together with decreased FtMt, is congruent with the hypothesis that tumor cells require more iron for growth than normal cells [59]. Considering this, we predicted that the overexpression of FtMt would inhibit proliferation in nervous tissue tumors. To confirm our hypothesis, we used the previously characterized FtMt-SH-SY5Y [18, 19] cell line as a model. We found that elevated FtMt indeed interfered with cell cycle progression. Therefore, we continued to test this model by investigating the mechanisms of neuroblastoma proliferation inhibition.

Our results show that overexpression of FtMt not only markedly inhibited SH-SY5Y cell growth (Fig. 1b–e), but did so through an apoptosis-independent mechanism (Fig. 2a). Importantly, overexpression of FtMt did not affect normal tissue growth and development (Table 1). It has been previously shown that Fe depletion can induce G1/S arrest [8, 60]. We confirmed that excess FtMt can likewise halt the cell cycle at G1/S by a flow cytometric assay. Further, we determined that this was likely the result of a deprivation of functional cellular iron, since the cells responded to iron deficiency by increasing TfR1 and IRP2 protein levels with a concomitant decrease in those of ferritin (Fig. 4). Consistent with this, we observed a decrease in the LIP, which is likely to hinder the maturation of essential iron-containing proteins, such as RNR. A significant decrease in RNR could then hinder DNA synthesis and thus halt the cell cycle.

Cdks, cyclins and Rb phosphorylation influence cell proliferation through control of the cell cycle. To investigate the effects of FtMt on tumor proliferation inhibition, we assayed these proteins’ expression by Western blot analysis or qRT-PCR. Increased FtMt resulted in decreases in cyclinD1 and Cdk2, a slight increase in cyclinE, but no change in Cdk4. As cell cycle progression is controlled by cyclin–Cdks complexes, it is not surprising that cell proliferation was hindered under these conditions. Normally, cyclinD1 forms a complex with Cdk4, while cyclinE binds with Cdk2, in both cases activating kinases which then phosphorylate the retinoblastoma susceptibility gene product, Rb, among other targets. Phosphorylated Rb can then release the transcription factor, E2F1, which in turn translocates to the nucleus where it mediates the transcription of a range of genes vital for S-phase progression [61]. It is also worth noting that we observed a decrease in the Cdk2–cyclinE complex in NB and NS (Fig. 3).

Myc is documented to play a role in tumor initiation and regulation of cell growth and proliferation. Inhibiting Myc function has been show to be a possible therapeutic strategy [62]. It has been reported that c-myc can repress the expression of H-ferritin and stimulate the expression of IRP2. This suggests that c-myc may be involved in regulating intracellular iron levels and which is essential in the control of cell proliferation [63]. We observed that excess FtMt causes the downregulation of c-myc, thereby inhibiting Myc pathway-driven tumor cell proliferation.

The tumor suppressor p53 and Cdk inhibitor p21^CIP1/WAF1^ are also important regulators of cell cycle. A number of studies have reported elevated levels of p53 protein following Fe depletion [6, 64]. In our study, overexpression of FtMt markedly upregulated p53 (Fig. 5). p21^CIP1/WAF1^, whose gene is tightly controlled by p53, binds to the cyclinE/Cdk2 complex, preventing pRb phosphorylation and therefore progression through G1/S transition. Interestingly, and paradoxically, when expressed at very low levels, p21^CIP1/WAF1^ is required for the assembly of cyclinD/Cdk complexes; downregulation of p21^CIP1/WAF1^ in tumor cells was found to lead to increased apoptosis [22]. It has been shown that p21^CIP1/WAF1^ mRNA can be markedly upregulated by Fe chelation via a p53-independent pathway [7, 65]. Meanwhile, it has also been shown that Fe depletion can decrease the levels of of p21^CIP1/WAF1^ protein [66]. Our results are congruent with these previous studies, as we observed a marked upregulation of p21^CIP1/WAF1^ mRNA together with decreased p21^CIP1/WAF1^ protein upon FtMt overexpression, suggesting that elevated FtMt can inhibit tumor cell proliferation by affecting p21^CIP1/WAF1^ levels through a p53-independent pathway.

PCNA acts as an enhancing factor for DNA polymerase δ and is elevated in early S-phase to support the high rate of DNA replication during cell cycle progression in eukaryotic cells [67]. As one would expect, tumor cells generally express higher levels of PCNA [67, 68] than normal cells. Expression levels of PCNA correlate positively with other pathological indices in prostate cancer and can serve as an independent prognostic marker. Upon overexpression of FtMt, we observed a decrease in the expression of PCNA, likely contributing to cell cycle arrest.

NDRG1, as a tumor suppressor, is negatively correlated with tumor progression and inhibits tumor cells metastasis in multiple neoplasms. Moreover, NDRG1 is an iron-regulated gene that is markedly increased by cellular iron depletion using iron chelators known to have anti-tumor properties [69]. However, the exact molecular function(s) of NDRG1 remain to be established and are important to elucidate. Our experiments show a slight decrease of NDRG1 in NB and NS compared to normal tissue. In contrast, excess FtMt significantly upregulated NDRG1 in SH-SY5Y cells, indicating that FtMt can have some effect on tumor cell growth and metastasis (Fig. 6).

Dependent on the cofactors Fe^2+^ and 2-oxoglutarate, JMJD1’s major function is to demethylate histone 3 (H3K9), resulting in transcriptional activation [41]. The possible contribution of JMJD1A deregulation to carcinogenesis is hinted at by some reports correlating the protein’s decrease with increased tumorigenesis [41–43]. Although we expected a decreased LIP to have some effect on this protein, we were unable to observe any significant influence of elevated FtMt on JMJD1A expression, indicating that either FtMt may not sufficiently sequester iron so as to inhibit the histone demethylase or that FtMt ability to block cell proliferation is independent of histone modification.

**Fig. 7:**
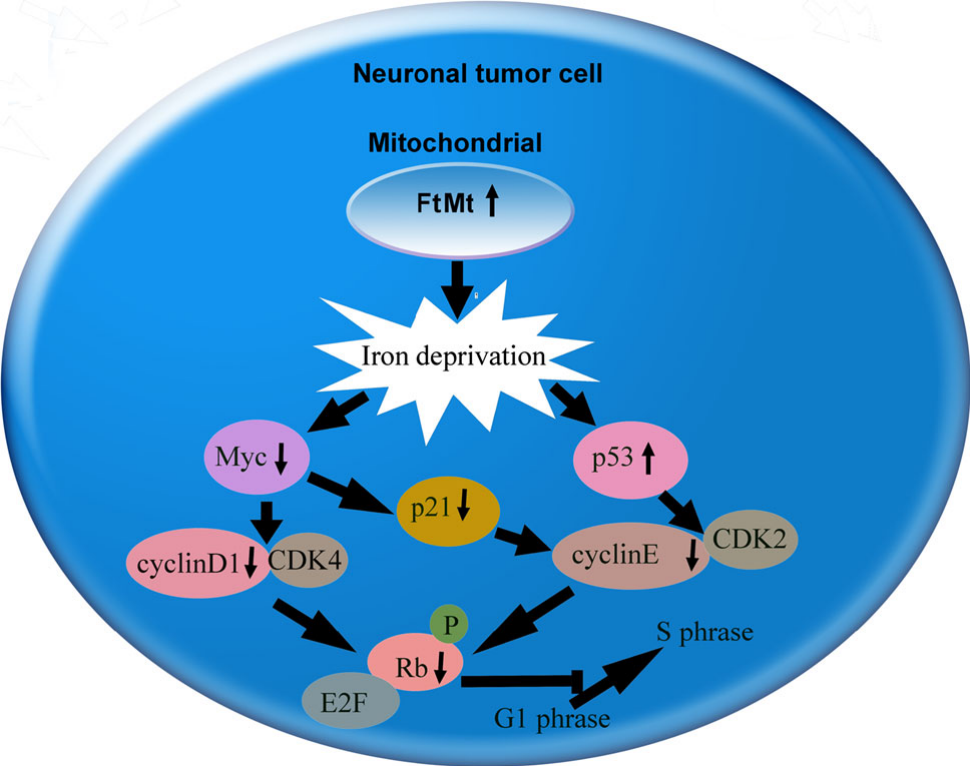
Model of the proposed mechanism of FtMt’s effect on inhibiting tumor cells growth. Under normal conditions, the progression of the cell cycle is controlled by cyclins, cyclin-dependent kinases, p53, p21, pRB/RB, c-myc and N-myc. However, high expression of FtMt caused cellular iron deprivation, and cellular iron deficiency caused the downregulation of Myc protein and upregulation of p53. Decreased Myc inhibited the complex formation of cyclinD1–CDK4 which caused the phosphorylation of Rb decrease. On the other hand, both decreased p21 and increased p53 inhibited the complex formation of cyclinE and CDK2, which also caused the phosphorylation of Rb decrease. Decreased pRb suppressed the release of E2F, thereby inhibiting some genes such as PCNA expression. In conclusion, FtMt caused cellular iron deprivation and inhibited cell proliferation by arresting the G1/S phase Meanwhile, iron deficiency upregulated NDRG1 which suppressed the metastasis of tumor cells

In summary, Fig. 7 shows a schematic representation of the proposed mechanism of FtMt as a new candidate target for inhibiting neuronal tumor cell proliferation.

### Electronic supplementary material

Below is the link to the electronic supplementary material.

Supplementary material 1 (DOC 51 kb)

## Abbreviations

Cdk: Cyclin-dependent protein kinase
CFSE: 5- or 6-(*N*-Succinimidyloxycarbonyl)-3′,6′-*O*,*O*’-diacetylfluorescein
FAC: Ammonium ferric citrate
FtMt: Mitochondrial ferritin
NB: Neuroblastoma
NBT: Normal brain tissue
NDRG1: N-myc downstream-regulated gene-1
NS: Neurospongioma
PCNA: Proliferating cell nuclear antigen
pRb: Phosphorylated retinoblastoma protein
Rb: Retinoblastoma protein
