# Supplementary material for: Mitochondrial ferritin, a new target for inhibiting neuronal tumor cell proliferation
Source: Cell Mol Life Sci. 2014 Sep 12;72(5):983–97. doi: 10.1007/s00018-014-1730-0 (PMC4323545; doi:10.1007/s00018-014-1730-0)
Supplement: Supplementary file 1 — Supplementary material 1 (DOC 51 kb) [file 18_2014_1730_MOESM1_ESM.doc]

| *Antibody Name* | *Catalogue Number* | *Company* | *Dilution* |
| --- | --- | --- | --- |
| **HA** | **sc-805** | **Santa Cruz** | **1:1000** |
| **β-actin**  **ferritin light chain** | **sc-130656**  **sc-390558** | **Santa Cruz**  **Santa Cruz** | **1:3000**  **1:1000** |
| **pRb** | **sc-16670** | **Santa Cruz** | **1:1000** |
| **Rb** | **sc-50** | **Santa Cruz** | **1:200** |
| **c-Myc** | **sc-40** | **Santa Cruz** | **1:500** |
| **N-myc** | **bs-5980R** | **Santa Cruz** | **1:200** |
| **mitochondrial ferritin** | **ab124889** | Abcam | **1:1000** |
| **ferritin heavy chain** | **ab65080** | Abcam | **1:5000** |
| **p21** | **ab109520** | Abcam | **1:1000** |
| **cyclinD1** | **ab101430** | Abcam | **1:1000** |
| **JMJD1** | **ab106456** | Abcam | **1:500** |
| **PCNA** | **ab140877** | Abcam | **1:1000** |
| **NDRG1** | ab37897 | Abcam | **1:1000** |
| p53 | MS-105-P0 | Thermo | 1:1000 |
| Cdk2 | MS-617-P0 | Thermo | 1:500 |
| Cdk4 | MS-616-P0 | Thermo | 1:200 |
| cyclinE  TfR1 | 630701  1348053A | Biolegend  Invitrogin | 1:200  1:2000 |

List of antibody name and catalogue number
